# Physics and applications of Raman distributed optical fiber sensing

**DOI:** 10.1038/s41377-022-00811-x

**Published:** 2022-05-07

**Authors:** Jian Li, Mingjiang Zhang

**Affiliations:** 1 College of Physics and Optoelectronics, Taiyuan University of Technology, Taiyuan, Shanxi 030024 China; 2 Key Laboratory of Advanced Transducers and Intelligent Control Systems (Ministry of Education and Shanxi Province), Taiyuan University of Technology, Taiyuan, 030024 China

**Keywords:** Imaging and sensing, Optical sensors

## Abstract

Raman distributed optical fiber sensing has been demonstrated to be a mature and versatile scheme that presents great flexibility and effectivity for the distributed temperature measurement of a wide range of engineering applications over other established techniques. The past decades have witnessed its rapid development and extensive applicability ranging from scientific researches to industrial manufacturing. However, there are four theoretical or technical bottlenecks in traditional Raman distributed optical fiber sensing: (i) The difference in the Raman optical attenuation, a low signal-to-noise ratio (SNR) of the system and the fixed error of the Raman demodulation equation restrict the temperature measurement accuracy of the system. {ii) The sensing distance and spatial resolution cannot be reconciled. (iii) There is a contradiction between the SNR and measurement time of the system. (iv) Raman distributed optical fiber sensing cannot perform dual-parameter detection. Based on the above theoretical and technical bottlenecks, advances in performance enhancements and typical applications of Raman distributed optical fiber sensing are reviewed in this paper. Integration of this optical system technology with knowledge based, that is, demodulation technology etc. can further the performance and accuracy of these systems.

## Abstract (web summary)

This paper review recent advances in Raman distributed optical fiber sensing in terms of temperature measurement accuracy, spatial resolution, dual-parameters and applications.

## Graphical abstract

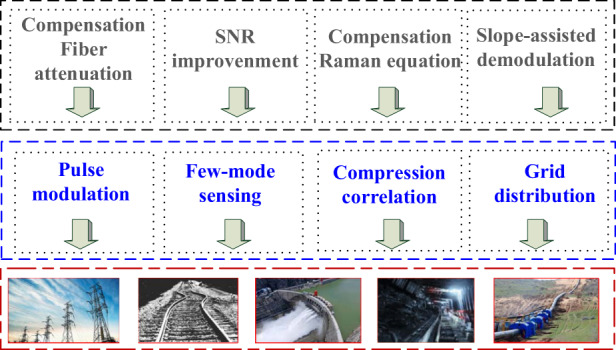

## Introduction

Distributed optical fiber sensors provide a method to measure the physical field of the surrounding environment through the distribution of different parameters, such as temperature^1,2^, strain^3–5^, vibration^6–8^, magnetic^9^ and gas sensing^10,11^, etc. across the sensing fiber. Owing to its detection ability, it has been widely used in micro-nano sensing^12^, medical treatment^13^, corrosive environment detection^14^, pressure sensing in harsh environments^15^, hydrophone sensors^16^ and other security detection fields. Based on the features of fiber scattering, the optical fiber sensing technology can be classified into Rayleigh fiber sensing, Brillouin fiber sensing and Raman fiber sensing. Among these, the Rayleigh optical fiber sensing is commonly used to detect attenuation characteristics^17,18^ and vibrations (phase optical time domain reflection sensing^19^) of the optical fiber. Brillouin optical fiber sensing can measure the temperature and strain distribution along the optical fiber^20–24^, and can obtain a high spatial resolution for long sensing distances. Raman optical fiber sensing can monitor a large-scale distributed temperature^25,26^. Compared to Brillouin scattering, the Raman scattering measurement system is not sensitive to strain, so there is no cross-sensitivity of temperature and strain information in it. However, owing to their fast measurement speed relative to other optical fiber sensors, and structural simplicity^26,27^, the Raman scattering sensors are receiving extensive attention^28–36^.

In 1928, physicist C. V. Raman, published a paper in *Nature*, titled, “A new type of secondary radiation”^37^. Subsequently, he won the Nobel Prize in physics for Raman scattering in 1930. Since then, the phenomenon of Raman scattering has been extensively and deeply studied, and many new Raman scattering effects have been discovered, such as spontaneous Raman scattering^38^, stimulated Raman scattering^39,40^ and resonance Raman scattering^41^. Currently, the Raman effect is widely applied in various disciplines, including physics, medicine, chemistry and biology^28–36,42–44^. Additionally, the principle of spontaneous Raman scattering is applied to developing distributed optical fiber sensors. The invention of lasers in 1960, followed by the establishment of the low-loss fiber theory in 1966, subsequent successful development of the first practical silica fiber in 1970, and the development of various new optoelectronic devices all promoted the development of Raman distributed optical fiber sensors^45,46^. In 1983, Dakin et al. first proposed to use the spontaneous Raman backscattered light to accomplish the distributed temperature measurement in theory^47^. Then the spontaneous Raman distributed temperature sensor was developed by his group in 1985^48^, which used an argon laser. A few years later, Hartog et al. used a semiconductor laser as a light generator to inject the sensing fiber-line, and consequently, developed the first sensor based on the principles of Raman scattering^49^. Subsequently, the Raman distributed optical fiber sensors have undergone rapid development^50–52^.

The recently developed Raman distributed optical fiber sensors are facing four theoretical or technical bottlenecks, which are discussed as follows. First, the difference in the Raman optical attenuation, limitation of the signal-to-noise ratio (SNR) and the fixed measurement error of the temperature demodulation equation, restrict the temperature measurement accuracy of the system. Second, the effective sensing distance and spatial resolution cannot be reconciled. Compressing the pulse width of the optical source can optimize the spatial resolution of the system, however, it will shorten the sensing distance of the system. In addition, owing to the limitations of the pulse time flight positioning method, the spatial resolution of a traditional long-distance Raman distributed optical fiber sensor is limited to the order of meters^53^. Most importantly, the spatial resolution will gradually deteriorate with an increase in the sensing distance^54^. Third, the SNR is inconsistent with the measurement time of the system. Increasing the cumulative average time of signals can effectively improve the SNR, however, the run time extends correspondingly^55^. Finally, Raman distributed optical fiber sensing is a temperature-based single-parameter demodulation technology, which cannot perform a dual-parameter, or multi-parameter cooperative detection. Considering these limitations, this article reviews recent advancements in Raman distributed optical fiber sensing principles and techniques.

The review article is organized as follows. The principle underlying Raman scattering and its limitations, and the schemes of temperature demodulation are explained and discussed in section “Principles and limitations”. The performances of temperature accuracy, sensing distance, spatial resolution and dual-parameter sensing are reviewed and summarized in section “Challenges and Methods”. Some typical applications of the sensing system are explained and discussed in section “Applications”. The scope for future development and its potential applications are discussed and analyzed in section “Trends”. The concluding remarks are summarized in section “Conclusion”.

## Principles and limitations

### Principles

Raman optical fiber sensing is based on the principle of Raman scattering, that is, a type of optical scattering where the interaction of a pulsed light with molecular motion changes the frequency of the incoming light when it passes through the sensing fiber^56^. The pulsed light either absorbs or emits optical phonons from or to the sensing fiber, subsequently, getting converted into an anti-Stokes light which has a high frequency, or a Stokes light with a lower frequency state, respectively^57^. The anti-Stokes and Stokes Raman photons, are expressed by Eqs. (1) and (2), respectively, as follows:

1 $$hv_s = h\left( {v_o - \Delta v} \right)$$

2 $$hv_{as} = h\left( {v_o + \Delta v} \right)$$

where *v*_*s*_ and *v*_*as*_ represent the frequency of the Raman Stokes and anti-Stokes signals respectively, *h* denotes the Planck constant, and *vo* is the initial frequency of the incident signal. The intensity of the Raman Stokes and anti-Stokes signals can be expressed using Eqs. (3) and (4), respectively, as follows:

3 $$\phi _s{{{\mathrm{ = }}}}K_s\mathop {\upsilon }\nolimits_s^4 \,\mathop {\Phi }\nolimits_o \left[ {1 - \exp \left( { - \frac{{h\Delta v}}{{kT}}} \right)} \right]^{ - 1}\exp \left[ {{\int}_0^L {\left( {\alpha \left( L \right) - \alpha _s\left( L \right)} \right){{{\mathrm{d}}}}L} } \right]$$

4 $$\phi _a{{{\mathrm{ = }}}}K_a\mathop {\upsilon }\nolimits_s^4 \,\mathop {\Phi }\nolimits_o \left[ {\exp \left( {\frac{{h\Delta v}}{{kT}}} \right) - 1} \right]^{ - 1}\exp \left[ {{\int}_0^L {\left( {\alpha (L) - \alpha _a(L)} \right){{{\mathrm{d}}}}L} } \right]$$

*K*_*a*_ and *K*_*s*_ represent the cross-section coefficient of the Raman anti-Stokes and Stokes signals, respectively. Δ*v*, k, *T*, and *L* denote the Raman frequency shift, Boltzmann constant, absolute temperature, position of the sensing fiber, respectively, and *αa*, *αs* and *α* are the attenuation coefficients of the anti-Stokes, Stokes and incident signals, respectively. Equations (3) and (4) show that changes in the surrounding temperature correspondingly change the modulation function of the Raman backscattering light. Furthermore, compared to the Raman Stokes backscattering light, the Raman anti-Stokes backscattering light is more sensitive to the external temperature^58^. Therefore, in the Raman anti-Stokes backscattering light is generally applied for temperature demodulation, and the Stokes light is simultaneously utilized as the reference light^59^. The typical temperature demodulation methods for Raman distributed optical fiber sensing are described as follows.

**Fig. 1:**
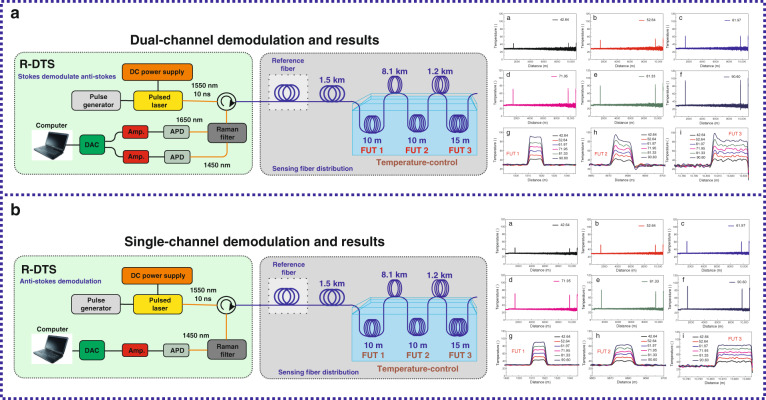
Experimental setup and results of the Raman distributed optical fiber sensor. **a** Experimental setup and typical results of the dual-channel demodulation principle. **b** Experimental setup and typical results of the single-channel demodulation principle.

Currently, the temperature demodulation methods are mainly divided into two types^36,47,60,61^, namely, the dual-channel demodulation technique and single-channel demodulation technique. The dual-channel demodulation technique uses the signal ratio of Raman Stokes intensity to the anti-Stokes intensity to detect the distributed temperature data^60,61^, while the single-channel demodulation technique only utilizes the Raman anti-Stokes signal to execute the self-demodulation process^36,47^. As shown in Fig. 1(a), the dual-channel demodulation scheme consists of a pulsed laser (1550 nm), a Raman filter (or a wavelength division multiplexer, 1550 nm/1450 nm/1650 nm), avalanche photodetector (APD), amplifier, data acquisition card (DAC), and a computer. When a pulsed laser is incidentally coupled to the optical fiber, a spontaneous Raman scattering occurs at all points along the sensing fiber, and the backscattered signals arriving at the Raman filter can be classified into an anti- Stokes (1450 nm) and Stokes signal (1650 nm). After that, these two optical signals enter the avalanche photodetector and amplifier for photoelectric conversion and amplification, respectively. Finally, the digital signal through the data acquisition card is transmitted to the computer for temperature demodulation processing. Figure 1(b) reveals that the temperature variation information distributed along the fiber can be accurately extracted through the dual-channel demodulation method. Conversely, the single-channel demodulation method uses the Raman anti-Stokes light to resolve the distributed temperature information along the optical fiber^36,47^, and the corresponding experimental device is depicted in Fig. 1(c). The results of the dual-channel demodulation method are illustrated in Fig. 1(d). Compared with the dual-channel demodulation system, the single-channel demodulation system only consists of an amplifier and an APD.

Both the abovementioned methods can theoretically detect the distributed temperature profiles across an entire optical fiber. Different temperature demodulation methods based on the application requirements. Despite the simplicity and ease of installation of the single-channel demodulation system, its pulsed source needs to be completely stable, and the attenuation properties of the sensing fiber must remain unchanged in the long-term temperature measurement process^62^. Due to this limitation, it has not been widely used in engineering applications. Contrarily, the dual-channel demodulation method uses the Raman Stokes light as a reference channel for demodulating the temperature so that the different loss coefficients can be corrected during the temperature demodulation process^60,61^. However, the SNR of the system becomes relatively poor after the introduction of the Raman Stokes signal^63^. The comparison of these two temperature demodulation techniques is presented in detail in Table 1^62–64^.

**Table 1:** Comparison of the dual-channel demodulation and single-channel demodulation principles

Schemes	Advantages	Characteristics
Dual-channel demodulation	(1) Eliminates the influence of optical source fluctuations. (2) It is suitable for engineering due to the stable measurement performance.	The fiber dispersion affects the measurement accuracy performance.
Single-channel demodulation	(1) The SNR and temperature accuracy performances are relatively better. (2) Simple device and easy to install.	The unstable light source affects its temperature accuracy performance.

### Theoretical limitations

Recently, because the temperature measurement scheme based on the Raman distributed optical fiber sensing can perform distributed detection and has the advantage of high stability, it has been extensively used in harsh environments^28–36^. The performance indicators for distributed optical fiber sensing mainly include temperature measurement accuracy, sensing distance, and spatial resolution^65–67^. However, the following theoretical or technical bottlenecks limit its sensing performance.

(i) The SNR of the Raman distributed optical fiber sensing system is weak compared to other optical fiber scattering signals, such as Rayleigh scattering and Brillouin scattering. This is because Raman scattering is a nonlinear effect, and the Raman scattered signal is about 60–70 dB weaker than the incident signal. This weak signal greatly limits the sensing performance of the system. Therefore, improving the SNR and extracting the weak Raman scattering signal is the key theoretical problem that needs to be addressed here. Moreover, the existing Raman scattering equations are not fully calibrated. The traditional Raman scattering transmission equation does not take into account the effects of the difference of optical attenuation, fiber sensitivity, fiber dispersion and other factors, which can also seriously affect the sensing performance of the system.
(ii) Sensing distance and spatial resolution cannot be reconciled. The traditional Raman distributed optical fiber sensing is based on the principle of optical time domain reflection for positioning, implying that the spatial resolution mainly depends on the pulse width. Compressing the pulse width can improve the spatial resolution, but it also limits the sensing distance of the system.
(iii) There is a discrepancy in the SNR and measurement time. The conventional Raman distributed optical fiber sensing needs to perform a large number of cumulative averaging to de-noise the scattered signal, but the cumulative averaging process will extend the measurement time of the system.
(iv) The Raman effect is only sensitive to surrounding temperature and cannot be effectively demodulated for other physical fields along the fiber-line, thereby, constraining it to a single parameter detection.

## Challenges and methods

**Fig. 2:**
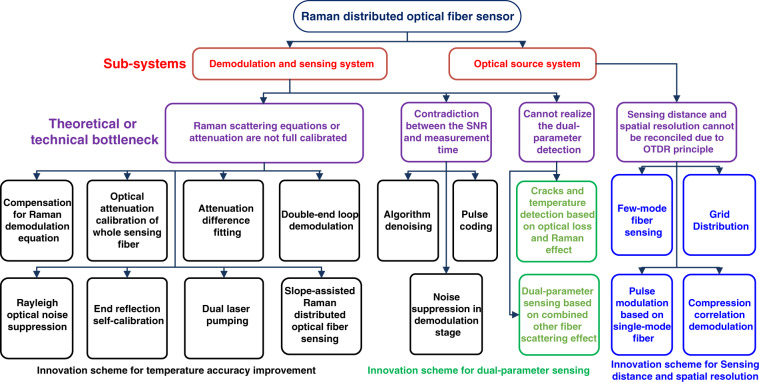
Advanced schemes of the Raman distributed optical fiber sensor. Among them, the red words represent the sub-systems. The black words represent the optimization scheme of temperature measurement accuracy, the blue words represent the optimization scheme of sensing distance and spatial resolution, the green words represent the dual-parameter sensing demodulation scheme, the purple words represent the theoretical or technical bottleneck of the system.

To satisfy the requirements of different engineering applications, researchers carried out some studies with the main purpose of developing high-performance Raman distributed optical fiber sensing, and explored various new theories and solutions to improve the performance of the system. This chapter introduces and summarizes the performance optimization of the sensing systems considering four aspects: temperature measurement accuracy, sensing distance, spatial resolution, and multi-parameter monitoring. Figure 2 presents the demodulation schemes for performance improvement of distributed optical fiber sensing. Its sub-systems mainly consist of the demodulation and sensing system, and the optical source system. The connecting lines represent the theoretical or technical improvement of the scheme based on the above key components. Next, we begin to explain in detail the rationale and experimental results of the various schemes.

### Improvement of temperature measurement accuracy

Temperature measurement accuracy is the key sensing index of the system, which denotes the deviation of the measured temperature from the actual temperature value. It can be determined by the standard deviation or uncertainty of the measured temperature^68^. The main reasons that affect the temperature measurement accuracy of the system include: (1) the optical attenuation difference between the Raman Stokes anti-Stokes signals, (2) the limitation of SNR, (3) the demodulation deviation of Raman transmission equation, and (4) the principle of optical time domain reflection causes the temperature signal in the spatial scale of the pulse width to be compressed into a point, and finally the temperature signal detected at this point is less than the actual temperature. In this case, researchers proposed and demonstrated a variety of advanced temperature demodulation programs to improve the temperature accuracy^68–117^. This section elaborates and analyzes the research progress pertaining to the improvement of the temperature measurement accuracy of the system.

#### Compensation for difference in the Raman optical attenuation

The traditional demodulation process used the intensity ratio of the Raman Stokes and anti-Stokes data to demodulate the ambient temperature information. However, the Raman Stokes and anti-Stokes signals exhibit a wavelength difference of about 200 nm when excited by an incident light of 1550 nm. Generally, in common single-mode fibers, the fiber attenuation of incident light at 1550 nm is 0.2 dB/km and that at 1310 nm is 0.4 dB/km^68^. Therefore, when this intensity ratio is directly used for temperature demodulation, a measurement error will inevitably be introduced., which is also the main reason for the failure of early systems in exceeding a sensing error of 1.0 km. So far, some advanced schemes have been demonstrated to compensate for this measurement error to improve the temperature accuracy^69–80^.

##### Dual laser pumping

**Fig. 3:**
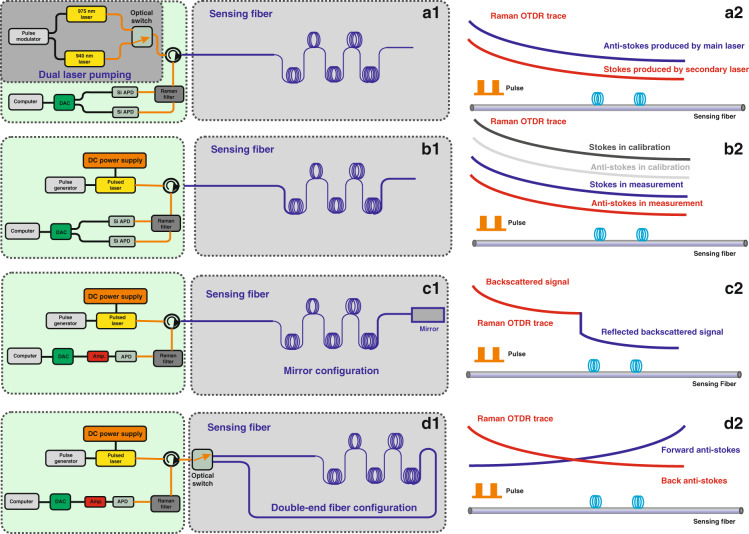
Advanced compensation schemes for attenuation difference proposed by researchers. **a1** Dual laser pumping scheme. **a2** Raman OTDR traces based on dual laser pumping. **b1** Optical attenuation calibration scheme and (**b2**) Raman OTDR traces based on the optical attenuation calibration scheme of the entire sensing fiber. **c1** End reflection self-calibration scheme. **c2** Raman OTDR traces based on the end reflection self-calibration. **d1** Double-end loop demodulation scheme. **d2** Raman OTDR traces based on double-end loop demodulation

As shown in Fig. 3(a1), Suh and Lee used two optical pulse sources of different wavelengths to generate the Raman backscattering signals^69^. Thus, we can obtain two signals with the same wavelength, thereby, achieving a consistency of the Raman optical attenuation coefficient. In the proposed scheme, the wavelength difference between the main laser and the secondary laser is twice the Raman frequency shift. In the experiment, the pulsed signals generated by the main laser and the auxiliary laser are alternately input through the optical switch, and thus, the anti-Stokes backscattered signal from the main laser and the Stokes backscattered signal from the auxiliary laser of the same waveband can be obtained, as shown in Fig. 3(a2). This method can eliminate the fiber dispersion effect introduced by the wavelength difference generated by a single laser, thereby, optimizing the temperature measurement accuracy performance. Therefore, it offers a higher measurement precision in Raman distributed optical fiber sensing. However, the above proposed method requires the main and secondary laser to possess a stable wavelength, resulting in an extra expense (secondary laser)^68^.

##### Optical attenuation calibration of whole sensing fiber

In addition to the influence of the attenuation difference between the Raman Stokes and anti-Stokes signal, the phenomenon of inconsistent attenuation along the entire length of the optical fiber also restricts the temperature accuracy performance^70^. Fortunately, the optical attenuation calibration of the sensing fibers can solve this problem, and the measurement setup is presented in Fig. 3(b1). The sensing fiber needs to be placed in a stable temperature environment prior to the demodulation process^71^. Subsequently, these Raman intensity data based on the constant temperature condition are used as the calibration data for the fiber attenuation coefficient, and the calibrated intensity is defined using Eq. (5). Following this, we can extract the distributed temperature information from the ratio of the Raman intensity obtained in the measurement phase (Eq. (6)) and the Raman signal obtained in the calibration phase, and the temperature demodulation curve is shown in Fig. 3(b2).

5 $$\frac{{\phi _{ao}}}{{\phi _{so}}}\frac{{\phi _{sco}}}{{\phi _{aco}}} = \exp \left[ { - \frac{{h\Delta v}}{k}\left( {\frac{1}{{T_o}} - \frac{1}{{T_{co}}}} \right)} \right]\exp \left[ {{\int}_{L_c}^L {\left( {\alpha _s(L) - \alpha _a(L)} \right){{{\mathrm{d}}}}L} } \right]$$

6 $$\frac{{\phi _a}}{{\phi _s}}\frac{{\phi _{sc}}}{{\phi _{ac}}} = \exp \left[ { - \frac{{h\Delta v}}{k}\left( {\frac{1}{T} - \frac{1}{{T_0}}} \right)} \right]\exp \left[ {{\int}_{L_c}^L {\left( {\alpha _s(L) - \alpha _a(L)} \right){{{\mathrm{d}}}}L} } \right]$$

*Φ*_*ao*_ and *Φ*_*so*_ represent the Raman anti-Stokes and Stokes intensities under the calibration condition. *Φ*_*ac*_ and *Φ*_*sc*_ denote the Raman anti-Stokes and Stokes intensities in the calibration fiber. *Φ*_*aco*_ and *Φ*_*sco*_ are the Raman anti-Stokes intensity and Raman Stokes intensity of the calibration fiber under the calibration condition. *L*_*c*_ is the position of the calibration fiber, *T*_*o*_ and *T*_*co*_ denote the calibration temperature of the sensing fiber and calibration fiber.

The above scheme is currently one of the most commonly used temperature demodulation methods for Raman distributed optical fiber sensing^72,73^, which requires a total of four whole-fiber Raman demodulation curves. However, this temperature demodulation method has a little drawback. When the sensing fiber is bent or broken at local positions while placing it at the scene of action, the optical attenuation coefficient will be inconsistent with the calibration stage, resulting in large temperature measurement errors, which severely limits the applicability of Raman distributed optical fiber sensors. Among the various schemes and methods that are applied to resolve the temperature measurement accuracy deterioration issue, arising because of an inconsistency in the optical attenuation coefficient, the three most typical schemes are the end reflection self-calibration scheme^74^, double-end loop demodulation scheme^75–79^, and attenuation difference fitting scheme^80^.

##### End reflection self-calibration

In 2010, Hwang and Seo et al. proposed an end reflection self-calibration scheme^74^, in which an end-face reflector was fixed at the end of the optical fiber-line; the experimental setup is shown in Fig. 3(c1). Compared with the traditional Raman distributed fiber sensing system, the proposed scheme can simultaneously acquire two types of Raman backscattered signals excited by a pulse signal in the sensing fiber, namely, the backscattered and reflected backscattered signals. The mirror reflects the forward Raman scattered signal to the entrance port of the fiber, and is subsequently, collected by the system, which can compensate for the influence of fiber attenuation. By demodulating the backscattering Raman anti-stokes signal and the Raman anti-Stokes scattered signal reflected by the end-face reflector, the temperature change along the fiber can be accurately extracted, as illustrated in Fig. 3(c2). Under these circumstances, the Raman signal reflected by the reflector is equivalent to the Raman signal in the calibration stage (optical attenuation calibration of the whole optical fiber-line), which can suppress the influence of optical attenuation. Most importantly, the traditional calibration method cannot solve the problem of sudden fiber loss; conversely, these two types of scattered signals obtained by the end reflection scheme are collected at the same time, which can avoid the influence of the sudden fiber loss during the measurement process.

##### Double-end loop demodulation

In the double-ended loop demodulation scheme, the sensing fiber is set into a loop configuration through an optical switch, and then the pulse laser is incident onto the front and end of the sensing fiber^75–79^. The experimental setup is presented in Fig. 3(d1), and the distributed temperature information is demodulated by detecting the forward and backward Raman backscattering signal, as shown in Fig. 3(d2). The use of these two different directions of the Raman backscattered signals for operation can solve the influence of the difference in light attenuation over time. The typical demodulation method is expressed using Eq. (7) as follows:

7 $$\begin{array}{l}R_{Loop}(T,L) = \sqrt {\frac{{\phi _a^B}}{{\phi _s^B}} \cdot \frac{{\phi _a^{_F}}}{{\phi _s^{_F}}}} = \frac{{K_a}}{{K_s}}\left( {\frac{{\upsilon _a}}{{\upsilon _s}}} \right)^4\exp \left( { - \frac{{h\Delta v}}{{kT}}\left( {M(L) + M(l - L)} \right)} \right)\\\qquad\qquad\quad {{{\mathrm{ }}}} \ast \exp \left[ {{\int}_0^l {\left( {\alpha _s(L) - \alpha _a(L)} \right){{{\mathrm{d}}}}L} } \right]\end{array}$$

The *Φ*_*a*_^*B*^ and *Φ*_*s*_^*B*^ are the Raman anti-Stokes and Stokes intensities in the case where the optical switch is connected to the end of the fiber. *Φ*_*a*_^*F*^ and *Φ*_*s*_^*F*^ are the Raman anti-Stokes and Stokes intensities in the case where the optical switch is connected to the front of the fiber. *L* is the length of the sensing fibber. *M*(*L*) and *M*(*l*_*L*) denote the modulation function related to the fiber temperature sensitivity.

In 2011, Soto and Bolognini et al. designed a double-end loop demodulation scheme based on Raman anti-Stokes signals^77^, achieving the temperature measurement accuracy of 1.1 °C at a sensing distance of 19.0 km. Moreover, in 2012, they used a double-ended loop demodulation device based on an anti-Stokes signal to obtain a temperature measurement accuracy of 0.43 °C along a 10.0 km long optical fiber-line^78^, and simultaneously analyzed the impact of the local optics loss on the system in detail. Whereafter, in 2016, Saxena et al. came up with the empirical mode decomposition-based double-end configuration to obtain the distributed temperature information^79^. Subsequently, in 2019, Li and Zhang et al. proposed a double-end loop demodulation scheme based on the reference temperature^72^, which realized a temperature accuracy of 1.2 °C along a 12.0 km optical fiber-line; its loop configuration is demonstrated in Fig. 3. Most importantly, the proposed scheme can avoid the pre-calibration stage, which can improve the applicability of the system. However, the double-end loop demodulation scheme requires a sensing fiber with twice the length of the sensing fiber to set up in a loop configuration.

##### Attenuation difference fitting

**Fig. 4:**
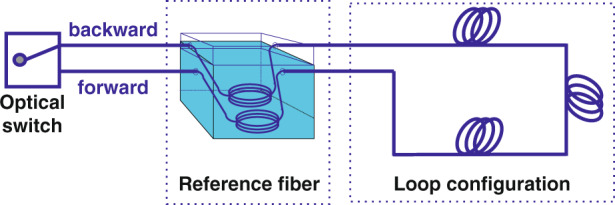
Loop configuration scheme using reference fiber in Raman distributed optical fiber sensor.

In 2020, Xia and Xia et al. showed that linear fitting and a calibration coefficient can correct for the Raman signal attenuation^80^. The key concept of the scheme is to calculate the distribution of the attenuation difference along the sensing fiber, and then confirm the calibration coefficient accordingly. They also validated that the processed and calibrated data exhibit a good linearity with the temperature at every sensing point. The enhanced system had a maximal measurement error of 1.56 °C at a distance of approximately 10.0 km (Fig. 4).

The above mentioned five advanced solutions solve the issues pertaining to achieving a good temperature measurement accuracy. Furthermore, the sensing performance can be estimated by evaluating the ratio of the sensing length to the temperature accuracy (sensing length/ temperature accuracy). This is because under the premise of a long sensing distance, the smaller the temperature measurement accuracy, the better its sensing performance. The advantages and characteristics of these five methods are shown in Table 2.

**Table 2:** Comparison and analysis of the compensation methods applied for obtaining a consistent Raman optical attenuation

Functions	Scheme Types	Advantages	Characteristic	Representative Results
Compensation for the optical attenuation difference between the Raman Stokes and anti-Stokes signal.	Dual laser pumping	The pre-calibration process is omitted, and the influence of fiber dispersion is eliminated.	An extra expense and complex structure for practical applications^80^.	/
Compensation for the attenuation changes with respect to the sensing distance.	Optical attenuation calibration of the sensing fiber	Simple operation, no additional devices.	Re-calibration is required after replacing any device or sensing fiber^72^. The sudden fiber loss cannot be eliminated.	0.36 °C at 10 km^72^ Figures of merit: 27.77
Compensation for the attenuation changes with respect to time.	Attenuation difference fitting	Eliminates the extra fiber loss due to fiber bending.	The sudden fiber loss cannot be eliminated.	1.56 °C at 10 km^80^ Figures of merit: 6.41
	End reflection self-calibration	Eliminates the extra fiber loss due to fiber bending.	SNR is lower than single-ended and double-ended demodulation^77,78^. Increased difficulty in data processing^80^.	2.95 °C at 4.2 km^74^ Figures of merit: 1.42
	Double-end loop demodulation	Avoids the influence of fiber bending on the measurement results. More suitable for engineering applications.	Requires twice the length of the sensing fiber and longer measurement time^80^. The SNR is optimal at the mid-position of the sensing fiber.	0.43 °C at 10 km^78^ Figures of merit: 23.25 1.10 °C at 19 km^77^ Figures of merit: 17.27

#### SNR improvement

Furthermore, the effective signal denoising method is also one of the core technologies applied in the Raman distributed optical fiber system for improving the SNR performance. To demodulate the distributed temperature data, the interference of noise must be eliminated. This is because the Raman stokes and anti-Stokes intensities are weaker than the Rayleigh scattering intensity in Raman distributed optical fiber sensors^81^. If this weak signal is used for temperature demodulation, it is bound to limit the performance of the sensing system. Besides, the system noise is generated in each step of the demodulation process. For instance, when the pulse is emitted from the laser and enters the sensing fiber, and when it returns to the detector after the occurrence of Raman effect in the optical fiber-line. The noise of the system mainly includes the optical noise (Rayleigh optical signal) and electrical noise. The noises generated in photodetectors and other circuit systems belong to electrical noises, mainly including multiplied scatter noise, system thermal noise, etc.^82^. The generated electrical noise exhibits zero-mean characteristics, and most of them belong to the Gaussian white noise category^83^.

**Fig. 5:**
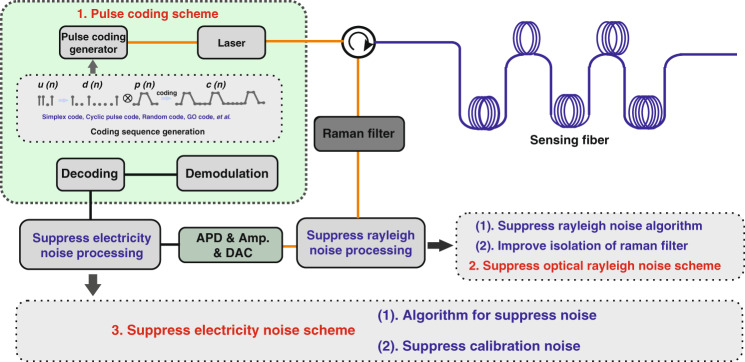
Various denoising schemes in improving temperature measurement accuracy performance. Among them, the pulse coding scheme can increase the luminous flux incident on the sensing fiber under the premise of suppressing the nonlinear effect of the fiber, thereby improving the SNR. Algorithm denoising, noise suppression scheme in the calibration stage, and the Rayleigh optical noise suppression scheme improve the SNR by suppressing the electrical noise and optical noise, respectively.

The simplest denoising method is to average the Raman backscattering signals collected by the system, which can suppress the electrical noises. However, increasing the average times of the system inevitably deteriorates the measurement of the system time. Consequently, the existing Raman distributed optical fiber sensor exhibits a contradiction between the measurement time and the SNR. To solve the aforementioned issue, a large number of advanced SNR optimization schemes have been used to improve the temperature accuracy performance. Based on the various techniques, it is mainly divided into pulse coding, algorithm denoising, Rayleigh noise suppression and noise suppression scheme in the demodulation stage. The flow diagram of the denoising stage is presented in Fig. 5, which explains the execution stage and stage of these several denoising schemes of the Raman distributed optical fiber sensing system.

##### Pulse coding

The detection pulse source is encoded and modulated in the proposed pulse coding scheme, and the corresponding decoding process is designed in the demodulation system to obtain the distributed temperature data^84–92^. In conventional Raman distributed optical fiber sensors, increasing the power of the pulsed laser can optimize the SNR. However, a nonlinear effect occurs when its optical power exceeds a certain threshold^85^. This phenomenon greatly limits the temperature demodulation results of the system. The pulse coding scheme can improve the SNR and dynamic range without increasing the pulse power^92^. Because the pulse coding scheme can increase the number of photons in the emitted signal without generating system nonlinear effects (such as stimulated Raman effect), it encodes the laser based on a functional equation before the laser is incident into the sensing fiber, and subsequently, decouples and restores the collected signal through the decoding program. In addition, the decoding and demodulation scheme can also effectively suppress the noise of the system. This is because the decoding system can effectively restore the Raman scattering signal excited by the coded laser through the decoding process, and the electrical noise of the system is effectively suppressed since, it has not been coded. Based on the above two reasons, the intensity of the Raman scattering signal is significantly enhanced, thereby, improving the SNR. Therefore, the pulse coding scheme is an ideal advanced solution to optimize the SNR of the system.

In 2004, Lee et al. applied a simplex coding scheme in a Raman distributed optical fiber system for the first time, and demonstrated an improvement of 4.5 dB in the SNR^84^. In 2011, Soto et al. used pulsed lasers to generate high-power pulse signals, and then used an EOM modulator to perform the 71-bit cyclic coding on the pulse signal. The experiment achieved a temperature measurement accuracy of 3 °C on the 26 km long single-mode fiber^85^. Simultaneously, the team used a laser based on Fabry-Perot cavity as the light source, a Mach-Zehnder modulator to modulate continuous light into pulsed light, and an acousto-optic modulator for simplex coding, and achieved a temperature measurement accuracy of 4 °C at a sensing distance of 58 km^86^. Subsequently, more advanced pulse coding technology is applied in Raman distributed optical fiber sensing, and the typical pulse coding schemes and measurement index are shown in Table 3. Coding technologies mainly incorporate simplex coding^87^, low-repetition-rate cyclic pulse coding^85^, cyclic pseudo-random sequence coding^89^, and pre-shaped simplex code^90^. Sun and Hong proposed and demonstrated a novel demodulation scheme in 2020^92^, which encoded the interrogation optical data using a single-sequence aperiodic code, and resolved the optical fiber data spatially through rapid demodulation processing. Moreover, a temperature measurement accuracy of 0.5 °C was obtained experimentally at a sensing distance of 40 km.

**Table 3:** Typical pulse coding schemes and experimental results

Schemes	Sensing distance	Temperat-ure accuracy	Figures of merit
255-bit Simplex Coded^87^	19.5 km	3.0 °C	6.5
Low-Repetition-Rate Cyclic Pulse Coding^85^	26.0 km	3.0 °C	8.66
Cyclic Pseudo-Random Sequence Coding^89^	1.0 km	1.5 °C	0.66
Pre-Shaped Simplex Code^90^	50.0 km	9.0 °C	5.55
Improved Simplex Coding^91^	1.0 km	0.1 °C	10
Genetic-Optimized Aperiodic Code^92^	39.0 km	3.9 °C	10

##### Algorithm denoising

With the development of digital signal processing technology, a variety of excellent algorithm denoising methods are used in Raman distributed optical fiber sensing, such as short-term Fourier transform^93^, dynamic sampling-correction scheme^94^, and wavelet denoising algorithm^95–97^. Among them, the short-term Fourier transform converts the time series of Raman signal timing into the frequency domain information, and subsequently, filters out some high-frequency noise to improve the SNR of the system. But this approach sometimes destroys the integrity of the signal. The wavelet denoising algorithm has been widely applied in the Raman optical time domain reflection system. It can not only optimize the SNR of the system without increasing the system measurement time, but also very suitable for denoising the optimization of distributed signals owing to its incapability of filtering out the sudden temperature signal of the system.

In 2015, Saxena et al. demonstrated a signal processing scheme based on wavelet denoising and applied it to the Raman distributed optical fiber sensing. The wavelet denoising method is an algorithm based on the multi-resolution analysis of wavelet transform. The wavelet coefficient of the noise is smaller than that of the signal. Wavelet coefficients larger than a reference threshold are considered to be generated by signals and need to be retained, and those smaller than the threshold are considered to be generated by noise and set to zero to achieve the purpose of denoising. The method could achieve a temperature accuracy of 3.5 °C^96^. Subsequently, in 2018, Li and Zhang et al. proposed and demonstrated an improved wavelet transform modulus maximum scheme combined with the dynamic difference denoising^97^ method. In this scheme, the signal behind the Fresnel reflection end in the Raman scattering signal is regarded as the base noise and the dynamic difference calculation is performed for signal denoising. The experimental results show that it can achieve a temperature measurement accuracy of 1.58 °C for a sensing distance of 10.4 km.

Thereafter, in 2019, Soto et al. used the non-local mean denoising method based on non-local self-similarity (NSS) of 2D images to the Raman distributed optical fiber sensing^98^, thereby, realizing a more remarkable denoising effect. This scheme is based on the characteristics of white noise and random noise, and exploits the high level of similitude and redundancy contained on the multi-dimensional information measured by distributed temperature signal. Furthermore, Zhang and Tang et al. proposed a deep 1D denoising convolutional neural network scheme to improve the temperature measurement accuracy of the Raman distributed fiber sensing in 2020, which made the temperature demodulation process of the system more intelligent and convenient. The proposed scheme can be understood as a filter, and it can achieve arbitrary filtering effects through multiple layers of convolution and nonlinear operations. By training a large amount of data, the system can gradually optimize its filtering parameters to achieve noise suppression. These experimental results achieved a 0.7 °C at sensing distance of 10 km^99^.

Recently, in 2021, Datta and Srinivasan et al. proposed and demonstrated the application of conventional total variational denoising filters to Raman distributed fiber sensing. This scheme presents a deep learning framework for multi-event detection with an enhanced measurement accuracy using the data measured from a Raman distributed fiber sensing system. It sanitizes the data using correlation filtering to remove any undesirable noise spikes before feeding it to a deep learning network to achieve multi-event detection. The experiment obtained a temperature accuracy of 0.5 °C at a sensing distance of 11.5 km^100^. Since, the algorithm denoising technology does not increase the hardware cost of the sensor, the temperature demodulation scheme based on the algorithm has attracted extensive attention from researchers in recent years. Some typical schemes based on algorithm denoising and its measurement index are shown in Table 4. Recently, some advanced algorithmic denoising schemes have been fully applied to the Raman distributed optical fiber sensing systems^93–105^.

**Table 4:** Typical algorithm denoising schemes and results

Schemes	Sensing distance	Temperature accuracy	Figures of merit	Year
Short term fourier transform^93^	0.23 km	0.50 °C	0.46	2014
Dynamic sampling-correction scheme^94^	20.0 km	1.00 °C	20	2015
Deconvolution algorithms^101^	30.0 km	2.70 °C	11.11	2015
Total variation deconvolution^103^	1.3 km	0.10 °C	13	2016
D-SVD algorithms^104^	0.60 km	0.87 °C	0.68	2019
Principal component analysis^105^	0.65 km	0.30 °C	2.16	2020
Deep learning^99^	10.0 km	0.70 °C	14.28	2020

##### Rayleigh optical noise suppression

The abovementioned algorithm denoising methods can effectively suppress the electrical noise of the system, without effectively removing the optical noise. In the Raman distributed optical fiber sensing, Raman filter is usually applied to split and filter the Raman backscattering signal in the optical fiber. However, the optical isolation of the Raman filter is about 38 dB, which cannot completely filter out the Rayleigh backscattering light^106^, which results in the accumulation of massive Rayleigh optical noise in the Raman anti-Stokes and Stokes signal. If this signal is used in temperature demodulation, it is bound to cause a large measurement error in temperature accuracy. Therefore, many advanced Rayleigh optical noise suppression schemes are applied in Raman distributed optical fiber sensors^107–109^.

In 2013, Sun and Chang et al. demonstrated a new demodulation scheme for improving the temperature measurement accuracy by suppressing the Rayleigh optical noise^107^. The principle is based on the sensitivity of Raman scattering in the fiber to ambient temperature, and insensitivity of Rayleigh scattering to ambient temperature. The authors place a section of reference fiber in the sensing fiber at two different ambient temperatures, and then measures a difference and performs logarithmic operations on the two sets of signals. The system can then calculate the Rayleigh noise amplitude for that segment of the reference fiber location. Finally, the Rayleigh noise amplitude of the entire sensing fiber is calculated through data fitting. The experimental results indicated that the Rayleigh optical signal occupies about 15% of the anti-Stokes channel. The temperature results proved that the temperature detection error can be suppressed from about 7 to 2%.

In 2014, Wang and Chang et al. proposed and demonstrated an adaptive temperature demodulation method, aimed at omitting the Rayleigh optical noise. The principle of temperature demodulation and Rayleigh noise suppression of this method is similar to that of the previous method. The difference is that this method is applied to the Raman anti-Stokes single-channel demodulation system, and the position of the reference fiber of this method is adjustable, which can be used to calculate the Rayleigh noise amplitude coefficient at different positions. The experiment demonstrated an effective suppression of the measurement errors^108^ and an improvement of temperature measurement accuracy and SNR of the system.

Furthermore, in 2019, Yan and Zhang et al. proposed a dual all-fiber calibration scheme for suppressing the Rayleigh optical noise, which can simultaneously upgrade the temperature measurement accuracy and temperature resolution of the system^109^. The authors believe that subsequent to the determination of the light source and system components, the Rayleigh scattering signal collected by the acquisition system along the fiber at any time is certain. Therefore, they placed the entire sensing fiber under two different ambient temperature conditions to collect the Raman scattered signals, and thereafter, used the two Raman OTDR traces to perform difference calculation methods to eliminate the influence of Rayleigh noise on temperature demodulation. Experimental results show that the proposed scheme optimizes the temperature measurement accuracy from 6.2 to 1.7 °C within a sensing distance of 9.1 km. Compared with the traditional demodulation scheme, the temperature resolution is improved by about 1.5 °C at a sensing distance of 10.0 km. The above-mentioned methods are used for algorithm demodulation to filter the optical Rayleigh noise. Because the optical Rayleigh noise is introduced by an insufficient isolation of Raman filter, improving the isolation of the Raman filter. However, this solution increases the cost of the system.

##### Noise suppression in the demodulation stage

**Fig. 6:**
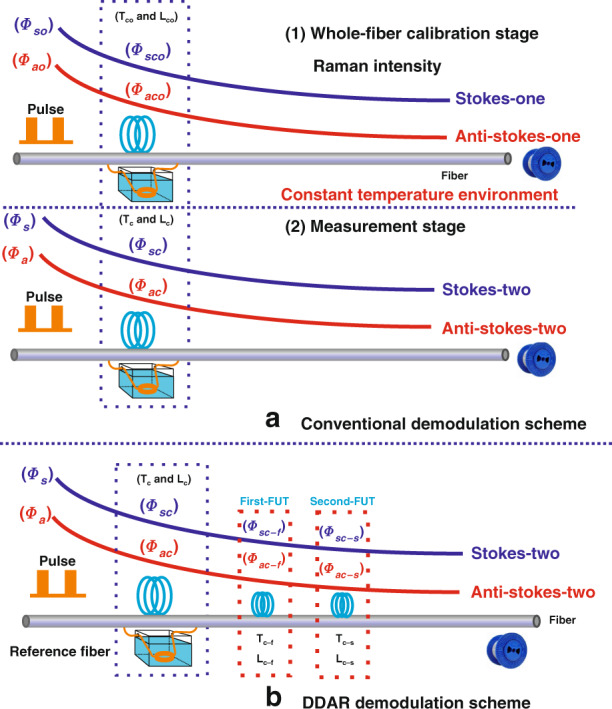
Demodulation principles and schemes of the distributed optical fiber sensors. **a** Schematic diagram of conventional demodulation scheme. **b** Schematic diagram of dynamic difference attenuation recognition scheme.

In the conventional Raman demodulation process, the sensor requires two parts of the fiber backscattered signal for temperature demodulation. As for the sources of Raman backscattering light intensity information, one is from Raman Stokes and Raman anti-Stokes signals in the measurement stage, and the other is from Raman Stokes and Raman anti-Stokes signals in the whole-fiber calibration stage before measurement. The conventional temperature process scheme requires the optical fibers to be placed in a stable temperature environment before measurement^110^. Thus, the conventional temperature demodulation scheme requires four-segment Raman OTDR trace for demodulation, as shown in Fig. 6(a). In this calibration process, a large amount of system noise is doped into the collected Raman signal channels, resulting in the decrease of SNR. When the sensor uses such demodulated signals with a low SNR to extract the temperature information in the optical fiber, a decrease in the temperature accuracy performance of the system is inevitable.

In 2020, a novel dynamic difference attenuation recognition principle and scheme in the Raman distributed fiber sensing was proposed by Li and Zhang^111^. The authors set up two reference fibers in the sensing fiber, and then calculated the attenuation coefficient based on the Raman scattering signals at the positions of the reference fibers, and collected the Raman OTDR traces for calculation. The temperature information along the sensing fiber can be demodulated based on the two-segment signal, as shown in Fig. 6(b). This can avoid the pre-calibration process of the conventional temperature demodulation scheme, thereby, suppressing the influence of the pre-calibration interference noise on temperature demodulation. The experiment results proved that a temperature accuracy with 0.18 °C was achieved at a sensing distance of 17.0 km using the dual-channel demodulation scheme. Furthermore, a temperature accuracy of 0.30 °C at a sensing distance of 17.0 km was achieved for the single-channel demodulation scheme. The proposed technology could optimize its SNR to 12.82 dB and 13.32 dB through the dual-channel demodulation and single-channel demodulation schemes, respectively.

The above-mentioned advanced schemes can improve the temperature measurement accuracy performance by optimizing the SNR; the function analysis and characteristics are shown in Table 5. Additionally, the abovementioned advanced schemes can be used in combination to further improve the sensing performance of the Raman distributed optical fiber sensing systems.

**Table 5:** Comparison and analysis of SNR improvement methods based on Raman distributed optical fiber sensing

Scheme Types	Advantages	Characteristic	Representative Results
Pulse Coding	It can effectively improve the SNR and sensing distance at the same time.	An extra expense and complex for practical application.	3.9 °C @ 39.0 km^92^, Figures of merit: 10; °C @ 1.0 km^91^, Figures of merit: 10;
Algorithm Denoising	Operation process is simple. Reduce the number of averaging and optimize the measurement time of the system.	Some useful signals may be filtered out.	2.7 °C @ 30.0 km^98^, Figures of merit: 11.11
Rayleigh Optical Noise Suppression	Operation process is simple.	It cannot eliminate electrical noise, and needs to be used in combination with other denoising methods.	1.7 °C @ 9.1 km^109^, Figures of merit: 5.35,
Noise Suppression in the Demodulation Stage	The calibration process is omitted, and operation is simple.	It needs to be used in combination with other denoising methods.	0.18 °C @ 17.0 km^111^, Figures of merit: 94.4

#### Compensation for Raman demodulation equation

The traditional Raman scattering equations, such as Eqs. (3) and (4), do not take into account the effect of some device compensation coefficients on the intensity modulation of the Raman scattering signal, such as the temperature sensitivity of APD and that of the sensing fiber. If the Raman scattering equation is not calibrated during temperature demodulation, it also seriously affects the temperature measurement accuracy of the system. Recently, many researchers have found that the fiber dispersion^112–114^, temperature drift of device^116^, and temperature-sensitive characteristics of the sensing fiber^63^ also affect the temperature measurement accuracy. In order to solve the above-mentioned problems, researchers further enhanced the temperature accuracy performance by modifying the Raman demodulation equation. In this section, we analyzed and reviewed these advanced solutions in detail.

##### Compensation for fiber dispersion

In the dual-channel demodulation system (Raman Stokes over Raman anti-Stokes signal), because of the fact that the Raman Stokes and anti-Stokes signals have different wavelengths, their propagation speed in the optical fiber is different. Therefore, these two types of signals which are scattered from the same position reach the photodetector at a different time, therefore, system cannot deduce that the two signals arrived from the same position^112^. The misalignment in position introduces temperature measurement errors in the temperature variation area. Furthermore, the measurement error is greater at the end of the sensing fiber due to the influence of fiber dispersion. The conventional correction method for fiber dispersion is to add a matching optical fiber of different length in the temperature measurement area to ensure a consistency in the transmission time of the two channels^113^, so as to eliminate the position error introduced because of the difference in optical speeds of the two signals. However, this method has certain limitations, because it can only compensate for a specific length, while the time difference between the two signals due to fiber dispersion can be generated at any point on the fiber line. In 2013, Wang and Chang et al. proposed a wavelength dispersion scheme based on the speed difference of the Raman Stokes and anti-Stokes light to compensate for this position error^114^. Besides, in 2019, Li and Zhang proposed and experimentally verified a new Raman distributed optical fiber sensing scheme based dual-reference-fiber^72^. The experimental results indicated that the temperature measurement accuracy could be optimized from 5.6 to 1.2 °C at a sensing distance of 13 km. The above methods employ multi-mode sensing fibers to compensate for fiber dispersion. However, if a single-mode fiber is used for distributed sensing, the influence of fiber dispersion on the temperature measurement accuracy of the system can be effectively suppressed.

##### Compensation for temperature sensitivity of APD

Because the Raman signal is extremely weak, it is necessary to use an APD detector to collect this weak signal in the Raman distributed optical fiber sensing system^115^. However, the performance of APD is easily affected by fluctuations in the surrounding temperature. When the surrounding temperature changes, the system noise, APD gain, and the multiplication factor of the detector also change, which eventually change the output voltage of APD, thereby, affecting the stability of temperature measurement.

**Fig. 7:**
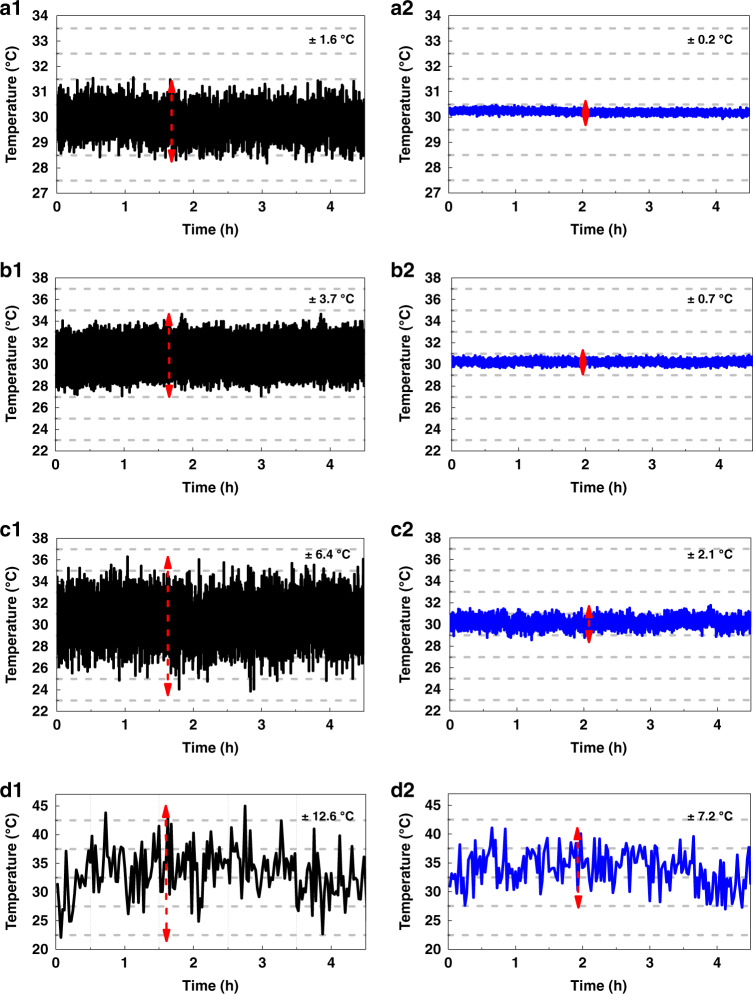
Measurement temperature stability at different sensing distances. Experimental results using the conventional demodulation scheme at (**a1**) 1.38 km, (**b1**)10.22 km, (**c1**)18.80 km, and (**d1**) 28.90 km. Experimental results at (**a2**) 1.38 km, (**b2**) 10.22 km, (**c2**)18.80 km, and (**d2**) 28.90 km using the auto-correction scheme. Reprinted with permission from ref. ^116^, © The Optical Society

In 2019, Li and Zhang designed and verified an auto-correction method to improve the temperature measurement accuracy^116^. The presented scheme can suppress the unstable photoresponsivity of the system. In the proposed scheme, a segment of reference fiber was placed in the optical fiber, and then, the intensity ratio of the measurement fiber and the reference fiber was used to suppress the influence of the light source and APD on the temperature sensitivity. Additionally, this paper applied a multi-stage constant temperature control to further reduce the operating temperature of the APD and simultaneously improving the SNR of the system, because with an increase in the gain of the APD, the operating temperature decreases. The operation temperature of APD could approach to 5.0 °C based on the multi-stage constant temperature control, and the demodulation method and typical experiment results are shown in Fig. 7 and described using Eq. (8); where *Φ*_sco_ and *Φ*_aco_ are the Raman Stokes and anti-Stokes intensities in the calibration stage, while *Φ*_sc_ and *Φ*_ac_ are the Raman Stokes and anti-Stokes intensities during the measurement stage. Experimental results indicated that the temperature accuracy of the multimode fiber was optimized from ±12.6 to ±7.2 °C within a sensing distance of 30.0 km, and the temperature accuracy of 2.1 °C could be obtained at a sensing distance of 18.80 km. Thus, it can be observed the proposed method is a viable solution when the automatic correction function is required to improve the performance of Raman distributed optical fiber sensing system.

8 $$T = \frac{1}{{\left( {\frac{1}{{T_c}} + \frac{1}{{T_o}} - \frac{1}{{T_{co}}}} \right) - \frac{k}{{h\Delta v}}\ln \left( {\frac{{\phi _{sco}\phi _{ao}\phi _{ac}\phi _s}}{{\phi _{aco}\phi _{so}\phi _{sc}\phi _a}}} \right)}}$$

##### Compensation for temperature sensitivity of the sensing fiber

The temperature sensitivity of the sensing fiber also affects the temperature measurement performance^63,117^. The temperature sensitivity gradually weakens with an increment of the effective sensing distance. Besides, the thermal insulation material of the optical cable also restrains the temperature sensitivity of the sensing fiber to some extent. Unfortunately, the traditional temperature demodulation process of Raman distributed optical fiber sensing does not take into account the influence of the temperature sensitivity of the sensing fiber. In 2020, a novel difference sensitive-temperature compensation scheme was proposed, which experimentally demonstrated an improvement in the temperature measurement accuracy^63^. It introduced a temperature sensitivity factor (*M*(*L*)) in the temperature modulation function. This factor can dynamically compensate the temperature modulation function of the Raman scattering equation, so that it can calibrate the temperature sensitivity of the sensing fiber at different sensing distances, and finally improve the temperature measurement accuracy of the system. The experimental results show that the temperature measurement accuracy could be optimized to 0.38 °C (in dual-channel demodulation), 0.36 °C (in single-channel demodulation) and 0.56 °C (in double-end loop demodulation) at a sensing distance of 10.0 km. The dual-channel demodulation, single-channel demodulation and double-end loop demodulation schemes based on the difference sensitive-temperature compensation can be expressed by Eqs. (9), (10) and (11), respectively^63^.

9 $$T = \frac{{M\left( L \right)}}{{\left( {\frac{{M\left( {L_c} \right)}}{{T_c}} + \frac{{M(L)}}{{T_o}} - \frac{{M\left( {L_{co}} \right)}}{{T_{co}}}} \right) - \frac{k}{{{{{\mathrm{h}}}}\Delta \nu }}\ln \left( {\frac{{\phi _{sco}\phi _{ao}\phi _{ac}\phi _s}}{{\phi _{aco}\phi _{so}\phi _{sc}\phi _a}}} \right)}}$$

10 $$T{{{\mathrm{ = ln}}}}\left\{ {\frac{{\left[ {\exp \left( {M\left( L \right)\frac{{h\Delta \nu }}{{kT_o}}} \right) - 1} \right]\left[ {\exp \left( {M\left( {L_c} \right)\frac{{h\Delta \nu }}{{kT_c}}} \right) - 1} \right]}}{{\left[ {\exp \left( {M\left( {L_{co}} \right)\frac{{h\Delta \nu }}{{kT_{co}}}} \right) - 1} \right]\left( {\frac{{\phi _a\phi _{aco}}}{{\phi _{ao}\phi _{ac}}}} \right)}} + 1} \right\}^{ - 1}\left( {M\left( L \right)\frac{{h\Delta \nu }}{k}} \right)$$

11 $$\frac{1}{T} = \frac{{M\left( L \right) + M\left( {l - L} \right)}}{{\left\{ {\left[ {M\left( L \right) + M\left( {l - L} \right)} \right]/T_o} \right\} - \left\{ {{{{\mathrm{l}}}}n\left[ {R_{Loop}\left( {T,L} \right)/R_{Loop}\left( {T_o,L} \right)} \right]\left( {k/h\Delta \nu k} \right)} \right\}}}$$

#### Slope-assisted Raman distributed optical fiber sensing

The Raman distributed optical fiber sensors are required to achieve accurate temperature measurements at a micro-scale. However, the demodulated temperature is the average temperature of the entire optical fiber section whose spatial length is equal to the spatial resolution, owing to the limitations of pulse width and the principle of optical time-domain reflectometry. Thus, the detected temperature is much smaller than the actual temperature when the detection area is smaller than the spatial resolution of the system. Li and Zhang et al. analyzed and demonstrated the pulse transmission feature in the temperature variation area and the superposition characteristics of Raman optical time-domain reflectometry signals through numerical simulations. Moreover, they proposed a slope-assisted sensing principle and scheme in a Raman distributed optical fiber system^57^. In this scheme, the falling edge of the superimposed Raman OTDR curve was defined as the slope-assisted detection area in the fiber under test (FUT). The temperature variation information along the FUT can be demodulated using the proposed slope-assisted coefficients.

**Fig. 8:**
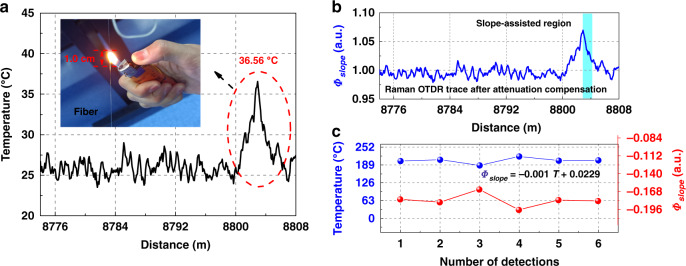
Temperature measurement results at a centimeter-level spatial scale detected using a conventional demodulation method and slope-assisted demodulation method. **a** Results measured using the conventional demodulation method, (**b**) distribution of superimposed Raman OTDR trace after attenuation compensation, (**c**) results measured using the slope-assisted coefficients. Reprinted with permission from ref. ^57^, © 2021 Chinese Laser Press

In the experiment, a cigarette lighter was used to heat the optical fibers, that is, it could maintain the temperature of the fiber up to a distance of 1.0 cm, as shown in Fig. 8(a); the core temperature of the flame can reach 200 °C. Limited by the spatial resolution, the temperature of the fiber was demodulated to 36.56 °C using a conventional demodulation method, as shown in Fig. 8(a). However, the conventional Raman demodulation method cannot accurately monitor the temperature in such a small area. Figure 8(b) shows the distribution of the superimposed Raman OTDR trace after compensating for attenuation. Figure 8(c) presents the slope-assisted coefficients (red point) and calculated temperature information (blue point). Among them, the slope-assisted coefficients were calculated in the slope-assisted area to be −0.18, −0.1845, −0.1645, −0.1966, −0.1812, and −0.1827 in six repetitive experiments, as shown by the red dots in Fig. 8(c). *Φ*_*slope*_ is the slope-assistant coefficient, which represents the descending rate of the Raman OTDR curve in the slope assistant region. *Φ*_*slope*_ is calculated from the Raman scattering intensity of any two adjacent points in the slope-assisted region. Based on the equation in Fig. 8(c), the corresponding demodulation temperature values were also calculated using the slope-assistant coefficients, which are 202.9 °C, 207.4 °C, 187.4 °C, 219.5 °C, 204.1 °C, and 205.8 °C, respectively, as shown by the blue dots in Fig. 7(c); the demodulated temperature results being consistent with the temperature of the flame. This is the first experimental demonstration of Raman distributed optical fiber sensing in a centimeter-level spatial measurement region.

### Improvement in the sensing distance and spatial resolution

Spatial resolution and sensing distance are also the key indicators of the effective sensitivity of Raman distributed optical fiber sensing systems^118^. First, the effective sensing distance that the system can achieve is related to factors such as the attenuation of sensing fiber, incident light power, and SNR of the system^119^. The light loss occurs when incident light is transmitted in the sensing fiber, and the intensity of the incident light gradually decreases with an increment of the effective sensing distance. As a result, the intensity of Raman backscattered light from the remote end of the sensing fiber is very weak, leading to the submersion of the Raman signal in the system noise and subsequent failure at effective extraction.

Owing to the types of fibers, the incident light and scattered signal undergo different losses during their transmission across the sensing fiber. Therefore, the optical fiber with a low-loss can effectively extend the sensing distance of the system. Also, increasing the incident light power of the pulsed light source increases the SNR of the system and ultimately extends the transmission distance^120^. However, due to the threshold characteristics of spontaneous Raman scattering, the incident light power cannot be increased indefinitely, and the sensing distance of the Raman distributed fiber is typically limited to about 10 km^68^. The Raman amplifier can further increase the energy of the incident light at the end of the fiber, thereby, extending the sensing distance of the system. For example, Kuznetsv and Kharenko et al. proposed an ultralong Raman distributed optical fiber sensing scheme which utilizes the standard single-mode telecom fiber and a 1.63-mm probe signal source, achieving a sensing distance of 85.0 km^121^.

The spatial resolution is defined as the minimum distance that the optical fiber sensing system can distinguish between two adjacent points. Moreover, the Raman distributed optical fiber sensing system is based on the OTDR system for positioning; therefore, the spatial resolution can also be expressed as the minimum fiber length that characterizes the resolution of the sensing fiber^122^. When the length of the FUT is less than the spatial resolution of the system, the measured temperature of the system is lower than the actual ambient temperature. Consequently, the spatial resolution can also reflect the minimum length of the sensing fiber-line required for the system to accurately measure the temperature. In case of Raman distributed optical fiber sensing, the detection signals are pulsed laser signals, and its theoretical spatial resolution mainly depends on the pulse width^54^. The best spatial resolution performance is achieved by the multi-photon counting technique, which provides a resolution of a few to tens of centimeters over sensing length of several meters. In a recent study, a spatial resolution with 1.0 cm over a sensing distance of 3 m has been proposed and demonstrated^123^, which is a significant improvement in terms of the spatial resolution, but is still far from practical applications considering the sensing distance^68^.

An additional bottleneck lies in balancing the sensing distance with the spatial resolution. Reducing the pulse width can optimize the system’s spatial resolution, but it deteriorates the system’s sensing distance. For example, Ososkov and Chernutsky et al. proposed and demonstrated a sensing scheme based on an ultrashort pulse mode-locked fiber laser^124^. Experimental results show that this scheme achieves a temperature resolution of 1.5 °C and a spatial resolution of 10 cm at an effective sensing distance of 3.0 m. Moreover, this ensures that the spatial resolution of the existing long-distance Raman distributed optical fiber system is to limited to the order of meters^89^. In addition, fiber dispersion widens the pulse width, and spatial resolution is further deteriorated with an increase of the sensing distance, resulting in a spatial resolution over several meters or even tens of meters.^53^. This phenomenon severely limits the application of the Raman distributed optical fiber sensing systems^80^. To optimize the sensing distance and spatial resolution performance of the system, researchers have proposed many advanced solutions, and the specific methods are discussed as follows.

#### Pulse modulation based on single-mode fiber

Currently, multi-mode fiber and single-mode fiber are used in Raman distributed optical fiber sensing as the transmission and sensing media, respectively. Compared with the multi-mode fiber, the single-mode fiber exhibits a lower transmission loss, and thus, is viable for remote sensing solutions^88^. Moreover, dispersion through the single-mode fiber is small, hence, its pulse width remains unchanged owing to a long-distance transmission, which can ensure that the spatial resolution does not gradually deteriorate with the increase of the sensing distance^54^. However, the small Raman gain of the single-mode fiber lowers the SNR of the Raman signal, which ultimately affects the sensing distance performance^88^. To improve the spatial resolution of the system based on single-mode fiber sensing and ensure a long sensing distance, researchers have encoded and modulated the light source^87,88,125–129^. And the proposed scheme can increase the luminous flux coupled to the sensing fiber as a result of suppressing the nonlinear effect of the fiber, thereby, increasing the effective sensing distance of the system^126^. Furthermore, the single-mode fiber ensures that the spatial resolution does not deteriorate with an increase in the length of the sensing fiber.

For instance, Park et al. used a light source modulation technology to achieve a spatial resolution of 17.0 m for a 37.0 km long sensing fiber^87^. The effective sensing points could simultaneously evaluate the system’s sensing distance and spatial resolution performance^129^. The calculation was based on the coefficient ratio of the sensing distance to the spatial resolution (sensing length/spatial resolution). This technical parameter, that is, the coefficient ratio can be seen as a figure of merit for the sensing distance and spatial resolution. The effective sensing points of this scheme were measured to be 2,176. Contrarily, Soto et al. used a 71-bit cyclic coding scheme to achieve a spatial resolution of 1.0 m for a 26.0 km long sensing fiber^88^, with the number of effective sensing points up to 26,000. Besides, Taki et al. used the cyclic pulse coding method to obtain a spatial resolution of 1.0 m for a sensing distance of 10.0 km^128^, with the number of effective sensing points up to 10,000. Additionally, in 2020, Sun and Yang et al. proposed and experimentally proved a genetic-optimized aperiodic code scheme^92^, wherein, a spatial resolution of 1.0 m was obtained for a sensing distance of 39.0 km, and with the number of effective sensing points up to 39,000, which is the maximum number of effective sensing points among the current experimental schemes. The schemes and typical experimental results based on the pulse modulation for Raman distributed optical fiber sensing are illustrated in Table 6.

**Table 6:** Performance index of the pulse coding scheme

Schemes	Sensing distance	Spatial resolution	Figures of merit
Pulse coding^87^	37.0 km	17.0 m	2176
Cyclic pulse coding^128^	10.0 km	1.0 m	10,000
71-bit cyclic encoding^88^	26.0 km	1.0 m	26,000
Genetic-optimized aperiodic code^92^	39.0 km	1.0 km	39,000

#### Few-mode fiber sensing

Compared with conventional multi-mode fiber sensing schemes, the few-mode fiber sensing scheme can suppress the dispersion of the sensing fiber by using the few-mode fiber as its optical transmission medium. Along with attaining a long sensing distance, the spatial resolution performance can be optimized at the end of the fiber^130,131^. Compared with the single-mode fiber, few-mode fiber has a larger mode field area and a higher nonlinear threshold, which can enable the coupling of a larger incoming optical power to the sensing fiber, thereby, increasing the SNR and further extending the sensing distance. Compared with multi-mode sensing systems, the few-mode fiber can achieve a quasi-fundamental mode transmission, which effectively eliminates the inter-mode dispersion, achieving a higher spatial resolution during long-distance transmission. In 2017, Ming et al. used a second-order few-mode fiber whose effective sensing points were 6,666 to suppress the fiber dispersion over a sensing distance of 20.0 km, and achieved a spatial resolution of 3.0 m^130^ Moreover, in 2018, He et al. developed and proposed a novel graded index few-mode fiber with a large effective area and low dispersion, and achieved a spatial resolution of 1.13 m at a sensing distance of 25.0 km^131^. The number of effective sensing points of this fiber was 22,123. It can be concluded that different types of sensing fibers can achieve different performance indicators for Raman distributed optical sensing.

Table 7 lists the measured parameters of the FUT associated with Raman distributed optical fiber sensing. Among them, the A_eff_ is the mode effective area, g_R_ is the Raman gain coefficient; these two parameters are directly related to the SNR of the system. The higher the values of these two parameters, (A_eff_ and g_R_), the better the SNR, sensing distance and temperature measurement accuracy of the system. α denotes the fiber attenuation, and is directly related to the effective sensing distance of the fiber. For example, the single-mode fiber exhibits the least loss at a wavelength of 1550 nm, and the corresponding effective sensing distance is longer. P_th_ is the power threshold to simulate Raman scattering. It represents the maximum incident power that a Raman distributed optical fiber sensing system can allow for the incident light to pass through the sensing fiber. This implies that the larger the parameter, the larger the incident light power allowed to pass, and the better the sensing performance of the system (SNR, sensing distance and temperature accuracy). Table 8 shows the sensing results^131^, advantages, and characteristics of different fibers, which indicate that different sensing fibers should be used depending on their applications.

**Table 7:** Measured parameters of the FUT

Fiber type	A_eff_ [um^2^]	g_R_ [m/W]	α [dB/km]	P_th_ [W]
Muti-mode fiber	190	6.0 × 10^–14^	0.246	14.2
Single-mode fiber	80	6.2 × 10^–14^	0.200	2.1
Few-mode fiber	106	6.1 × 10^–14^	0.225	6.8

**Table 8:** Sensing results, advantages and characteristics of different types of sensing fiber schemes

Schemes	Sensing distance	Spatial resolution	Temperature accuracy	Advantages	Sensing characteristics
Multi-mode fiber sensing	25 km	2.58 m	0.7 °C	Larger backscattering coefficients, and its SNR is higher at an effective sensing distance. It is suitable for the fields which demand a high temperature measurement accuracy, such as petrochemical temperature monitoring, pipeline leakage fields.	(1) The fiber dispersion and attenuation coefficient deteriorate the effective sensing distance. (2) It is easy to stimulate the Raman effect.
Single-mode fiber sensing	25 km	1 m	6.9 °C	The fiber attenuation and fiber dispersion are small. It is suitable for long-distance linear engineering monitoring.	The Raman gain within the effective sensing distance is small. Temperature accuracy is small compared to other sensing schemes.
Few-mode fiber sensing	25 km	1.13 m	1.0 °C	The system combines the advantages of the multi-mode fiber scheme (high Raman gain) and the single-mode fiber sensing scheme (small fiber dispersion).	Few-mode fiber is more expensive compared to other sensing fibers.

#### Compression correlation demodulation

**Fig. 9:**
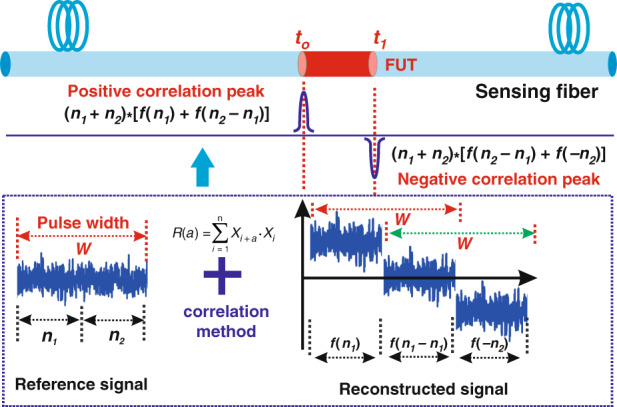
Schematic diagram and demodulation principle of the compression correlation demodulation scheme.

**Fig. 10:**
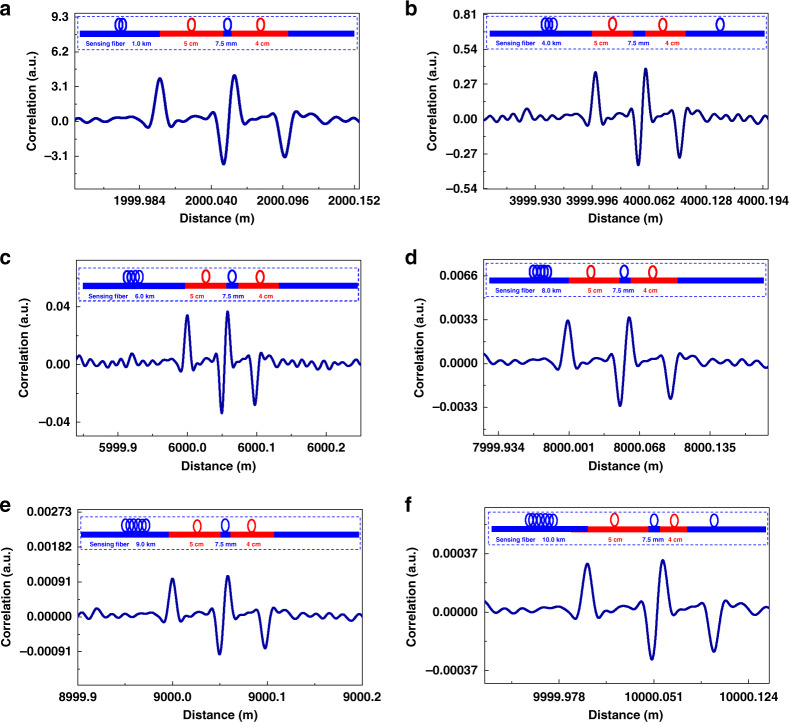
Spatial resolution results based on compression correlation demodulation^54^. **a** 2.0 km (**b**) 4.0 km (**c**) 6.0 km (**d**) 8.0 km (**e**) 9.0 km (**f**) 10.0 km. Reprinted with permission from ref. ^54^, © 2021 The Authors. Advanced Photonics Research published by Wiley-VCH GmbH

In 2021, Li and Zhang et al. proposed a compression correlation demodulation scheme aimed at improving the spatial resolution^54^. In the proposed scheme, an amplified spontaneous emission (ASE) source was used as the detection optical signal to replace the pulse source of traditional Raman distributed optical fiber sensing. The system then begins to reconstruct and decouple the Raman anti-Stokes signal excited by the ASE signal. This establishes a correlation between the reconstructed Raman anti-Stokes signal and ASE incident signal in the time domain. Finally, temperature change along the optical fiber line is obtained through the compression correlation demodulation scheme. The schematic diagram of compression correlation demodulation is shown in Fig. 9. This scheme uses the correlation method to compress the Raman anti-Stokes signal carrying the temperature change information in the FUT into the correlation peak to realize temperature demodulation. Here, the simulation results demonstrated a spatial resolution of 7.5 mm with number of effective sensing points up to 1,333,333, as shown in the Fig. 10. This is the maximum number of effective sensing points that can be achieved in the proposed Raman distributed optical fiber sensing scheme, despite the results being obtained in a numerical simulation system. The author claimed that the spatial resolution is independent of the sensing distance.

In addition, the spatial resolution of the compression correlation demodulation system depends on the full width at half height of the autocorrelation function of the detection signal. In order to further improve the spatial resolution of the system, Zhou and Zhang et al. proposed a chaos Raman distributed optical fiber sensing scheme^50^. A spatial resolution of 5 mm was achieved using a chaotic laser with 0.05 ns (full width at half height of the autocorrelation function) through the compression correlation demodulation scheme.

#### Grid distribution

In 2019, Zhang and Jin reported a novel 2D positioning method to dramatically improve the positioning accuracy and spatial resolution^132^. They achieved a 0.8 m spatial resolution along the 3.0 km multimode fiber using the method of demodulation of the Raman anti-Stokes signal by the Raman Stokes signal. And a positioning accuracy of about 0.1 m can be obtained without hardware modification. However, the sensing length is compromised owing to the grid distribution. This solution can be used on flat and curved surfaces, such as pipes or tank surfaces.

Among the advanced solutions mentioned above, the pulse modulation method and the few-mode fiber sensing method can improve the spatial resolution performance over long sensing distances. However, the theoretical spatial resolution of these two methods is still limited by the pulse width. At present, the spatial resolution achieved by experiments is limited to the order of meters^89^. The compression correlation demodulation method can achieve a millimeter-level spatial resolution performance, which is independent of the sensing distance. However, the compression correlation demodulation scheme requires the bandwidth of the APD with -50 dBm sensitivity to reach up to the GHz-level^54^. Therefore, the realization of high spatial resolution based on the compression correlation demodulation scheme needs to be further studied. Table 9 lists the advantages and characteristics of the above-mentioned schemes.

**Table 9:** Advantages and characteristics of different schemes for simultaneous improvement of sensing distance and spatial resolution

Schemes	Advantages	Characteristics	Figures of merit
Pulse modulation based on single-mode fiber	It can ensure a better spatial resolution along with extension of the sensing distance.	(1) An extra expense (EOM and coding generator) and operational complexity for practical application. (2) The theorical spatial resolution is limited to the pulse width, which is currently on the order of meters^89^.	39,000^92^
Few-mode fiber sensing	It combines the advantages of multi-mode fiber and single-mode fiber sensing.	(1) It is more expensive. (2) The theorical spatial resolution is limited to the pulse width, which is currently on the order of meters^89^.	6,666^130^ 22,123^131^
Compression correlation demodulation	Spatial resolution can reach the order of millimeters, and is independent of the sensing distance.	(1) An extra expense (ASE signal generator) for practical application. The bandwidth of the current commercial APD is difficult to meet its requirements.	1,333,333^54^ (Simulation)
Grid Distribution	(1) It does not require additional devices. (2) It is suitable for some applications.	(1) The spatial resolution is not improved in principle. (2) A longer sensing optical fiber setting network distribution is required.	3, 750^132^

### Dual-parameter sensing

At present, in the field of modern industrial monitoring, there is a strong demand for dual-parameter or even multi-parameter collaborative detection. For example, the distributed optical fiber sensing is required to realize the collaborative real-time monitoring of distributed temperature and structural cracks in tunnels^133^. Besides, the field of pipeline monitoring requires the dual-parameter detection of vibration and temperature along the optical fiber. Unfortunately, traditional Raman distributed optical fiber sensing is a single-parameter detection technology based on Raman scattering, which is unable to meet these requirements. In this case, development of a dual-parameter detection scheme based on a single optical fiber becomes an important technical problem for Raman distributed optical fiber sensing. For the purpose of solving the above-mentioned problems, researchers have proposed a variety of advanced solutions^26,134–138^, which are as follows.

#### Cracks and temperature detection based on optical loss and Raman effect

In 2019, Li and Zhang et al. demonstrated a novel dual-parameter optical fiber sensing scheme based on Stokes optical loss and the Raman effect to measure the distributed temperature and structural cracks^26^. The scheme was based on the double-end loop configuration and Stokes optical loss to extract the distributed temperature and crack information along the fiber-line. The experimental results attained a temperature accuracy of 0.28 °C and spatial resolution of 1.2 m under the response time of 1.04 s. Additionally, the detection range of the cracks was measured to be 1.6–5.6 mm with a resolution of 0.4 mm, while ensuring a high-precision temperature measurement. Subsequently, the Raman Stokes loss characteristics based on the optical time domain reflectometry curve were used to extract the crack information. The experimental results show that the Raman Stokes optical-loss coefficient can maintain a good linear relationship. This scheme can improve the applicability of Raman distributed optical fiber sensing systems. Most importantly, it proved that the temperature and crack could be measured using a single sensing fiber.

#### Dual-parameter sensing based on combined optical fiber scattering effect

**Fig. 11:**
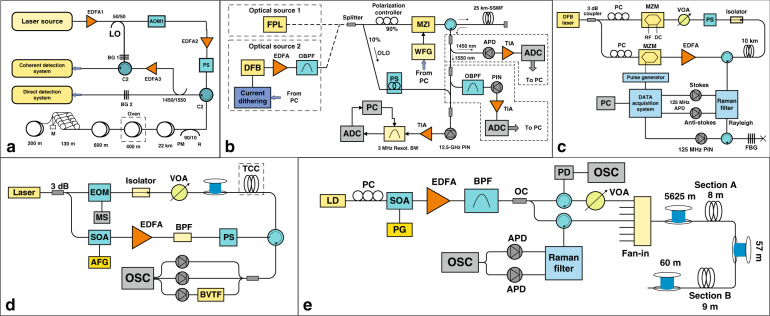
Experimental setup and results based on dual-parameter sensing. **a** Combined Brillouin and Raman scattering sensing, (**b**) hybrid Raman and Brillouin scattering sensing based on standard Fabry–Perot lasers in conjunction with optical pulse coding techniques, (**c**) hybrid Raman/Brillouin optical time-domain analysis of optical fiber sensors, (**d**) hybrid quasi-single mode operated Raman-Brillouin system in few mode fibers, (**e**) hybrid optical fiber sensor combined with the Raman optical fiber sensing and phase-sensitive optical time-domain reflectometry

Brillouin distributed fiber sensor can mutinously detect temperature and strain along the optical fiber, but faces the problem of cross sensitivity of temperature and strain^134^; contrarily, the Raman scattering signal is not sensitive to strain, so the combination of Raman and Brillouin scattering signals can perform the coordinated detection of temperature and strain along the fiber-line. In 2005, for the first time, Alahbabi and Cho et al. proposed to combine Brillouin and Raman scattering to simultaneously detect temperature and strain along the optical fiber^135^, and the experimental setup and results are shown in Fig. 11(a). In this scheme, the simultaneous measurement of temperature and strain along the fiber was achieved by using one optical fiber. It uses the Raman scattering effect to monitor the distributed temperature, and uses the Brillouin coherent detection system to measure strain along the fiber. In 2010, Bolognini et al. proposed and demonstrated the simultaneous measurement of strain and temperature based on hybrid Raman and Brillouin scattering. The proposed scheme uses a combination of standard Fabry-Perot lasers and optical pulse coding technology^136^. As such, the final temperature/strain resolution of approximately 0.27 °C/30 µε was attained over a 25 km sensing fiber range, as shown in Fig. 11(b). Subsequently, in 2013, Taki et al. experimentally demonstrated the hybrid Raman/Brillouin distributed optical fiber sensors for simultaneously detecting strain and temperature^128^. In this scheme, only a single narrowband laser source was used to measure strain and temperature at the meter level simultaneously over a standard single-mode fiber of 10 km, as shown in Fig. 11(c). Furthermore, a method for simultaneous distributed measurement of curvature and temperature was proposed and experimentally proved by Wu and Tang et al. This method utilizes the hybrid quasi-single-mode of the Raman-Brillouin system in a few modes fiber^137^. It achieves a spatial resolution of 1.5 m over a 2.0 km long optical fiber. The least obtained resolution of the square of the fiber curvature was 0.333 cm^–2^, and the temperature resolution of the fiber end was 1.301 °C, as shown in Fig. 11(d). In 2018, Zhao and Tang et al. proposed and demonstrated a hybrid optical fiber sensor that realized simultaneous sensing of the distributed acoustic and the distributed temperature^138^. The hybrid optical fiber sensor is constructed using a space division multiplexing reflectometer in a multi-core optical fiber, in which the Raman distributed optical fiber sensing and the phase sensitive optical time domain reflectometry are simultaneously realized through space division multiplexing. It obtained a temperature accuracy of 0.5 °C within a sensing range of 5.76 km, as shown in Fig. 11(e).

## Applications

The Raman distributed optical fiber sensing systems have the following advantages in terms of their applicability: (1) An optical fiber, which serves as the sensor and signal transmission channel, has the advantages of anti-flammability, anti-corrosion, and strong anti-electromagnetic interference ability, and can operate in extreme environmental conditions where traditional temperature sensors cannot work normally^139^. (2) A large number of point sensors can be replaced by an optical fiber, which can accurately measure the temperature information at any point along the direction of propagation; moreover, the sensing fiber exhibits high reliability and high feasibility for the distributed measurement and prediction of temperature at a low cost of production^140^. (3) The optical fiber is soft, light in weight, and easy to install^141^. As a transformative optical technology, the Raman distributed optical fiber sensor has been widely employed in a wide range of application domains owing to its unique characteristics^142–164^. These specific applications are discussed as follows.

### Fire-detection

Replacing a large number of point-type fire detectors with a temperature-sensing optical fiber to detect the distributed fire greatly reduces the number of components of the fire detection system, fully improves system reliability and effectively extends the system’s trouble-free time. The optical cables employed in Raman distributed optical fiber sensing can detect temperatures of 450 °C, and can even reach as high as 1000 °C when special sensing fiber is used^142^, since, the measurement range is determined by the sensing fiber characteristics^143^. Table 10 presents the effective range of temperature measurement of some special optical cables. Owing to its abovementioned advanced characteristics, the Raman distributed optical fiber sensor has been widely used in the temperature detection of some linear infrastructures, such as tunnels, and underground pipe corridors^144–146^. However, due to the meter-level spatial resolution of these sensors, the location of fire points on a smaller scale is still being studied. For example, in 2016, Sun et al. proposed a localization method for the detection of fire source using a Raman distributed optical fiber sensor and obtained good results^147^. In 2017, they proposed another location method for positioning the fire source^148^. The above two methods both use optical fiber folding mechanism to perform location detection of small-scale fire sources. However, the proposed method has some shortcomings in terms of its prediction accuracy and applicability.

**Table 10:** Fiber-based temperature measurement range

Optical fiber coating	Temperature range
Acrylate	–50 °C to 85 °C
Fluoropolymer	–50 °C to 200 °C
Thermoset Resin	–50 °C to 300 °C
Doped Metal	–50 °C to 550 °C

The speed of early warning reflects the response time of the sensor to sudden temperature changes, which is essential for fire monitoring. The early warning mode of traditional Raman distributed optical fiber sensing operates in real-time, and the early warning speed depends on the collection speed of the system^149^. To improve the forewarning time of the system, a direct way is to reduce the cumulative average times of the system, but this will result in the deterioration of the SNR, whereby, the early warning mode of the optical fiber sensor needs to be transformed from real-time early warning mode to the advanced early warning mode. Whereafter, the sensing fiber was found to have a certain temperature hysteresis effect when detecting the surrounding temperature along the sensing fiber, which prolonged the warning-time of the fire detection of the sensor. Thus, Li and Zhang proposed and experimentally demonstrated a Raman distributed optical fiber sensor based on the heat transfer functional model to perceive the surrounding temperature changes in advance^150^. The experimental results showed that the temperature change rate of the optical fiber maintained a linear relationship with the surrounding temperature difference after fitting, and this fitting relationship were used to perceive the surrounding temperature anomalies along the sensing fiber. The experimental results also indicated that the warning-time can be optimized from 23.4 to 1.3 s. To further improve the early warning speed of the system, this team proposed and verified a temperature early warning model for fire detection and prevention based on long-range Raman distributed optical fiber sensing^149^. As shown in the experimental results, the proposed scheme was able to predict the temperature trend in advance by 46 s. Additionally, the mean absolute error of the predicted temperature was 0.33 °C. Both these schemes provided effective solutions to improve the early warning time of fire monitoring.

### Pipeline leak detection

Pipeline leakage can not only result in the loss of resources, but also contribute to environmental pollution. In addition, the leakage of flammable and explosive products such as oil and natural gas may even give rise to fires and explosions. Therefore, the real-time monitoring of pipelines, timely detection of leakage, and prediction of hidden dangers are very important^151^. In case of pipeline monitoring, one or several optical cables are placed within the pipeline being laid. The optical fiber is used as a sensor to pick up the temperature signal around the pipeline, and further analysis and processing of this temperature signal can enable an accurate and quick determination of a leakage^152,153^. When the pipeline leaks, the liquid coming out of the pipeline will simulate a change in the temperature of the surrounding materials. Based on the OTDR positioning principle, the information of the leak location may be ascertained by the temperature signal detected by the sensing system.

Currently, distributed optical fiber temperature sensors are widely applied for determining pipeline leakage. For example, in 2017, Yamate et al. used these sensors for oil exploration and other fields such as downhole oil and gas fields, resulting in an accurate detection of the integrity of the oil and gas wells^154^. Thereafter, Apperl et al. used the Raman distributed optical fiber sensing system to locate and detect the leakage of sewage pressure pipelines^155^. Moreover, taking into account the temperature change characteristics of the pipeline leakage area, and combining it with the working characteristics of the Raman distributed optical fiber temperature sensors, Xu and Zhang et al. proposed a dynamic threshold detection method, in 2020^156^. This can effectively locate the leak by analyzing the variation in the ambient temperature around the leakage. In addition, Raman distributed optical fiber sensing can provide a more reliable guarantee for safe mining, safe storage and safe transportation in the field of oil logging monitoring. Recently, the Sensornet of the United Kingdom, the Schlumberger of the United States, and the Micron optics company are worldwide leading the application of Raman distributed optical fiber sensing in the field of oil and gas logging.

### Power system detection

As the transmission carriers of the power system, the cables are directly related to the safety and stability of the power grid. Currently, the hidden troubles of power cables, high-power voltage motors and other equipment are often accompanied by temperature rises in key parts of the equipment, which implies that overheating monitoring is an effective method for fault warning. With the development of the optical fiber technology, the application of Raman distributed optical fiber sensing in power equipment detection has been gradually promoted^157^.

A safe operation of the power cable is mainly ensured by monitoring the operating temperature of the cable conductor. Nevertheless, for power cables operating under high voltages, it is difficult to directly monitor the temperature of the running cable conductors. Thus, real-time monitoring of the temperature of the cable sheath through the distributed optical fiber temperature system is used to monitor the running status of the cable. For example, the Raman distributed optical fiber sensor installed on the high-voltage cable can perform real-time monitoring over long distances and alarm for faults in temperature along the power cable. Furthermore, the temperature of the generator bus in the end winding area needs to be continuously monitored. Therefore, Pelegrin et al. presented a reconstruction algorithm to obtain high-resolution signals, and readings of the distributed temperature using the sensor to monitor the high-power generator bars^158^. The purpose of this work is to use Raman distributed optical fiber sensing to measure the temperature of the end winding area. In addition, the proposed scheme makes it possible to use Raman distributed optical fiber sensing for temperature collection of small, medium and large motors. In 2020, Datt and Mamidala et al. reported an experimental and application demonstration of a Raman distributed optical fiber temperature sensor for real-time power line monitoring of overhead transmission cables^159^. Experimental research shows that this scheme can effectively monitor the distributed temperature of the cable sheath and conductor, and can effectively prevent and alarm the fault operation of the cable.

### Civil engineering monitoring

Owing to the complexity of civil engineered structures, it is difficult to measure the distributed temperature of the entire structure. Traditional temperature sensors based on electrical monitoring have a high temperature measurement accuracy, but exhibit a poor anti-interference ability and are complex in terms of installation. Fortunately, the distribution characteristic of the Raman distributed temperature sensor is very suitable for temperature monitoring of large civil structures. The applicability of these sensors to the field of civil engineering is as follows.

#### Dam structure

Traditional electronic sensors which have high production costs are extremely prone to errors during measurements, resulting in an ineffective monitoring^160,161^, whereas, the Raman distributed optical fiber sensing can safely monitor the leakage of a dam. The advanced optical sensing can monitor the temperature field of the dam and feedback the information of the structure’s leakage field, so as to complete the detection and location of the fault within the structure. For instance, Beck et al. used the Raman distributed optical fiber sensing to analyze the monitored temperature data of the dam, realizing the seepage and leakage monitoring of the dam^160^. Furthermore, Henault et al. conducted experimental researches on dam leakage detection and concrete structure strain monitoring based on the distributed optical fiber technology, simulating the strain between the optical fiber and the concrete structure^161^. Studying the location, direction and routing method of the sensing optical fibers in the dam are fundamental issues concerning dam leakage monitoring, and some researchers have elaborated on the application of optical fiber sensing instruments and their installation in actual projects^162–164^. Currently, with the improvement of various performance indicators of the optical fiber sensing technology, its applicability in the field of dam safety monitoring has become more extensive. In case of the Birecik dam in Turkey, a distributed optical fiber was laid at the bottom to monitor the distributed temperature of the entire dam, and the obtained data were accurate and reliable. Additionally, these experiments were carried out on a dam of China’s Three Gorges Project, realizing a real-time monitoring of the temperature field.

#### Tunnel structure

Owing to the compact structure of a tunnel, the occurrence of a fire hazard can cause major economic losses; hence, the early-warning monitoring of temperature distribution for the tunnel is extremely important. The Raman distributed optical fiber system can effectively measure the continuous distributed temperature field along the tunnel-line by taking into account its distinct structural environment. Yan and Zhang et al. proposed that the Raman distributed optical fiber sensors can be applied for a high precision measurement and visual positioning of tunnel fire detection^82^. Therefore, they proposed a temperature demodulation method to improve the temperature measurement accuracy of the sensors for this particular application. In addition, the longitudinal lining model of 3D temperature display was proposed, further enhancing the effectivity of the optical fiber scheme for tunnel fire detection. This tunnel monitoring network was designed via an industrial computer and Modbus. With the performance advantages of distributed optical fiber sensing, the application prospect of the monitoring system including temperature sensing will be of high value in the future.

## Trends

Raman distributed optical fiber sensing is a novel comprehensive sensing technology. Throughout the process of research, development and application, it is always used in some special industrial fields, especially in harsh environments and remote areas owing to its superior performance characteristics such as, anti-electromagnetic interference, long-distance detection, etc. Despite being studied and developed for nearly half a century, the sensing system still faces several theoretical and practical issues. To meet the demands of a wider range of practical applications, based on the principle of sensing and the current requirements, the main trends in the development of Raman distributed optical fiber sensing in the future include the following aspects.

### Extend the sensing distance

**Fig. 12:**
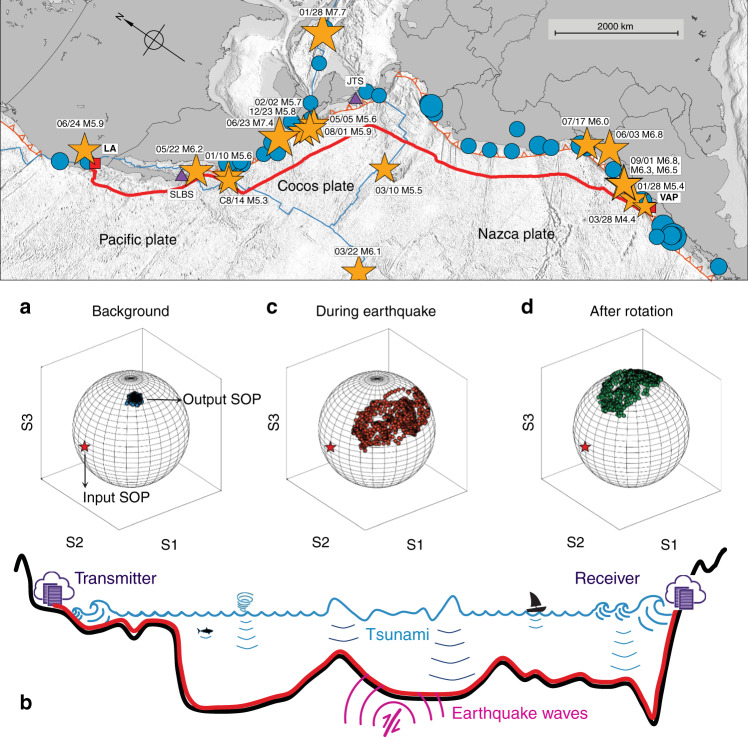
Ocean monitoring performed by exploiting the long sensing distance characteristic of optical fiber sensing. **a** The state of polarization (SOP) at the receiver is monitored routinely (blue dots on the Poincaré sphere) while the input SOP stays stable (red star). For the Curie cable, the output SOP is robust, owing to relatively minimal perturbations along most of its path in the deep ocean (**b**). The robustness allows us to detect earthquakes or ocean waves that produce SOP anomalies by shaking or pressuring the cable (**c**). Because the three Stokes parameters are normalized to 1.0, only two are independent. In this study, we rotate the Stokes parameters to the north pole of the Poincaré sphere (9) and focus on analyzing the S1 and S2 parameters after rotation (**d**).

Limited by the relatively weak SNR of the Raman backscattered signal, the effective sensing distance of the current commercial Raman distributed optical fiber sensor is mostly maintained at 10.0 km^53^, and some advanced systems can reach up to 30.0 km or 50.0 km. Furthermore, most distributed optical fiber sensors based on Brillouin scattering can reach up to 100.0 km^165^. In actual engineering applications, the sensing fibers are not laid linearly, rather a mesh to cover the blind spots of monitoring^166^, whereby, a longer sensing distance is required to lay the fiber. Unfortunately, the sensing distance of the existing Raman-based distributed optical fiber sensing system still cannot satisfy these requirements, resulting in the need for further optimization of its sensing distance performance. Therefore, in 2021, Zhan and Cantono et al. used optical fiber sensing to detect the seismic and water waves along the 10,000-kilometer submarine cable connecting Los Angeles. Related research results are published in *Science*^167^, and parts of the results are shown in Fig. 12. If the effective sensing distance of the Raman distributed optical fiber system is more than 10,000 km, the system can be used to detect the temperature of the ocean along the optical cable, which will play a vital role in the development of mankind. Therefore, it is important to develop new sensing mechanisms and methods to extend the sensing distance of the Raman distributed optical fiber sensors.

### Multi-parameter measurements

Engineering applications require the Raman optical fiber sensors to combine multiple optical fiber sensing schemes, along with the measurement of the various physical quantities, including temperature, strain, vibration, etc.^135–138^. Currently, the system mainly combines Brillouin and Rayleigh scattering to enable multi-parameter detection using one multi-core fiber or multiple sensing fibers. The disadvantage of the proposed combined structure is that it is relatively complicated. Therefore, there is an urgent need to develop a novel scheme of optical mechanism to achieve multiple physical field detection using a sensing fiber.

### Improvement of data processing

The current data processing algorithms for Raman distributed optical fiber sensing consider that the system noise is white noise, implying that the remaining noise of the system cannot be effectively removed. Contrarily, the crosstalk of multiple algorithms not only affects the measurement time of the system, but also eliminates part of the effective temperature variation signals, contributing to the phenomenon of underreporting of the system. Thus, novel data processing algorithms such as, the chaos algorithm^168,169^ and smarter deep learning algorithms based on optical fiber^170^ need to be developed.

### Intelligent networking

Currently, the Raman distributed optical fiber sensor should (1) perform functions such as condition monitoring and disaster warning, (2) be enabled to be used in multi-position, multi-parameter, and multi-purpose measurement occasions, (3) be capable of forming an intelligent optical fiber sensor network with dynamic real-time monitoring. With regards to further development, it is indispensable to integrate the optical fiber sensing technology with the knowledge-intensive technologies such as the computer network technology and information and communication technology. Alternatively, adaptive or deep learning algorithms can be applied to the system to automatically create a model consistent with the application environment, making the system more intelligent, and improving the practicability and adaptability of the system.

## Conclusion

In this paper, we have given a review on recent progresses in terms of the performance enhancement and applications of the Raman distributed optical fiber sensors. This work is concentrated on a retrospect of four aspects of Raman distributed optical fiber sensing, namely, the temperature accuracy, spatial resolution, sensing distance and multi-parameter measurements. Specific engineering applications of the sensing system such as, fire monitoring, pipeline leak detection, fault detection in power systems and structures such as dams have also been discussed and detailed. In summary, this review aims to clarify the current challenges, theoretical limitations and corresponding solutions of Raman distributed optical fiber sensing, and intends to provide some schemes to break through its limitations for practical applications.
